# 

**Published:** 1964-01

**Authors:** 


BRISTOL MEDICO-CHIRURGICAL JOURNAL

*(Incorporating the Medical Journal of the South-West)*

Established 1883

VOL. 79 (i) January 1964 NO. 291

### Contents

| | | |
|---|---|---|
| CAVERS DYING OF COLD . . . . . . | *O. C. Lloyd* | 1 |
| ADRENOCORTICAL HYPERFUNCTION AND OAT-CELL CARCINOMA OF THE BRONCHUS . | *D. Mattingly et al.* | 6 |
| TWENTY YEAR FOLLOW-UP OF A CASE OF MASSIVE CALCULUS IN A VESICAL DIVERTICULUM . . . . . . . | . *A. W. Adams* | 15 |
| CHLORPROPAMIDE IN PARKINSON'S DISEASE | *P. C. Barrington* | 16 |
| REPORTS FROM SOCIETIES . . . . . . . . | | 20 |
| BARROW HOSPITAL . . . . . . . . | | 21 |
| SOUTHMEAD HOSPITAL . . . . . . . | | 22 |
| NOTES AND NEWS . . . . . . . . | | 23 |
| APPOINTMENTS . . . . . . . . . | | 23 |

*PUBLISHED QUARTERLY*

*ANNUAL SUBSCRIPTION* 16/- *POST FREE*

PUBLISHED UNDER THE AUSPICES OF THE
BRISTOL MEDICO-CHIRURGICAL SOCIETY

### *Editorial Committee*

Dr. N. J. Brown, Southmead Hospital, Bristol. *Joint Editor.*

Dr. A. B. Raper, Department of Pathology, Bristol Royal Infirmary. *Joint Editor.*

Dr. J. Macrae, Ham Green Hospital.

Dr. R. C. Wofinden, Central Clinic, Bristol.

Mr. A. E. S. Roberts, Medical Library, University of Bristol. *Secretary.*

### *Local Correspondents*

*Bath* . . Mr. A. D. Bateman, 3 The Circus, Bath.

*Exeter* . . Dr. L. N. Jackson, Mount Jocelyn, Crediton, Devon.

*Gloucester* . Dr. R. F. Jarrett, 9 College Green, Gloucester.

*Plymouth* . Dr. C. R. Croft, Lexden, Hartley Av., Plymouth.

*Taunton* . Dr. M. McAlley, 1 The Crescent, Taunton.

*Torquay* . Dr. A. Robinson Thomas, 20 Devon Square, Newton Abbot, Devon.

*Truro* . . Dr. F. D. M. Hocking, St. Andrews, Perranporth, Cornwall.

*Yeovil* . . Mr. J. A. Vere Nicoll, Knapp House, Yeovil, Somerset.

### NOTICE TO CONTRIBUTORS

Original articles are invited, provided they have not been published elsewhere. Notes of forthcoming events, meetings held, degrees conferred, staff appointments, and other local news are welcomed. The editorial committee reserves the right to make literary corrections.

Illustrations should always be supplied ready for reproduction. All re-touchings or other operations required will be charged to the author. Line drawings should be drawn in Indian ink. We regret that we must ask authors to pay for making blocks, if this is necessary.

J. W. ARROWSMITH LTD., WINTERSTOKE ROAD,
BRISTOL.

### *Reports from Societies*

### WESSEX CLINICAL PATHOLOGISTS

The Branch met at the Bristol Royal Infirmary on 9th November 1963. Papers were read describing the following subjects: A family presenting the inheritance of two abnormal haemoglobins (Dr. A. B. Raper), the effect of bacteriostatic treatment on the bacteraemia following operations on the urinary tract (Prof. W. A. Gillespie), the isolation of an *Actinobacillus* causing chronic endocarditis (Dr. R. G. Mitchell), and the differential diagnosis of hypercalcaemia (Dr. M. Wills). Dr. Wills demonstrated that hyperparathyroidism could be distinguished from all other types of hypercalcaemia by the fact that in it the plasma chloride was raised above 102 mE/litre; this method of distinction was superior to the classically accepted changes in the plasma phosphate level.

### BARROW HOSPITAL

### WINTER LECTURES 1963-4

on

### Current Topics in Psychiatry at the Large Lecture Theatre, Queen's Buildings, University Walk, Bristol, at 8.30 p.m. on the following Thursday evenings:

### Programme

16th January: A. W. Beard, M.A., M.R.C.P., D.P.M. Physician in Psychological Medicine, The Middlesex Hospital. **"Current Problems and Trends in Schizophrenia".**

20th February: P. Sainsbury, M.D., D.P.M. Director of Research, M.R.C. Clinical Psychiatric Research Unit, Graylingwell Hospital. **"Evaluating Community Care".**

12th March: J. D. Pollitt, M.D., M.R.C.P., D.P.M. Physician in Psychological Medicine, St. Thomas's Hospital, London. **"The Functional Shift in Depression".**

2nd April: F. Post, F.R.C.P., D.P.M. Physician, Bethlem Royal and Maudsley Hospitals. **"Family Neurosis and Family Psychosis—a reappraisal".**

---

These lectures are open to medical practitioners, medical undergraduates, and others working in the field of psychiatry.

### BRITISH MEDICAL ASSOCIATION
### (BRISTOL DIVISION)

| | |
|---|---|
| Wednesday 15th January 1964 | 8.30 p.m. Lecture-Discussion. **'Early Diagnosis of Congenital Abnormalities'.** |
| Wednesday 27th May 1964 | 8.00 p.m. Clinical Meeting, Southmead Hospital Bristol. |

### *SUNDAY MORNING POST-GRADUATE MEETINGS FOR GENERAL PRACTITIONERS AT SOUTHMEAD HOSPITAL*

Meetings are informal. They are held in the Post-Graduate Lecture Room in the New Block (adjacent to T ward), and commence at 10.45 a.m. Coffee 10.30 a.m.

### *PROGRAMME*

1964

19th January: **"Open House".** General Practitioners will present their own cases for discussion.

26th January: Dr. H. G. Mather: **"Hypertension and its Treatment".**

2nd February: Clinico-Pathological Conference.

9th February: Dr. Ian Bailey: **"A Physician's Morning".**

16th February: Dr. Sarah Walker: **"The Health Authority, the General Practitioner and the Hospital".**

23rd February: Dr. A. T. M. Roberts: **"Asthma".**

1st March: Mr. A. G. McPherson: **"Surgical Treatment of Peptic Ulceration: Assessment and Results".**

8th March: Mr. C. A. Brown: **"The Ophthalmoscope and Some Common Eye Conditions".**

15th March: Dr. R. Harman: **"Dermatology".**

22nd March: **"Open House".**

### *Notes and News*

On his retirement as consultant in orthopaedic and traumatic surgery, Mr. N. L. Capener has been appointed emeritus consultant by the South Western Regional Hospital Board in recognition of his services to orthopaedics in the Devon and Exeter clinical area.

*M.R.C.P.:* P. C. Barrington, Aileen B. Donnison, P. R. Golding.

### *Appointments*

| | |
|---|---|
| G. V. Catford | Consultant Surgeon in Ophthalmology, St. George's Hospital. |
| C. I. Phillips | Consultant Surgeon in Ophthalmology, St. George's Hospital. |

SOUTH WESTERN REGIONAL HOSPITAL BOARD
CONSULTANTS, SPECIALISTS AND REGISTRARS APPOINTED DURING THE PERIOD JANUARY TO JUNE, 1963

### *BRISTOL*

| *Name* | *Appointment* |
|---|---|
| Abbas, T. M., M.D., (Edin.), F.R.C.S., M.R.C.O.G. | Consultant Obstetrician and Gynaecologist Bristol Clinical Area. |
| Bailey, I. S., B.SC., M.D., (Manchester), M.R.C.P. (London). | Consultant Physician, Bristol Clinical Area. |
| Buchanan, I. F. M., M.B., B.CHIR. (Camb.), D.OBST.R.C.O.G. | Clinical Assistant in Obstetrics, Weston-super-Mare General Hospital (2 sessions). |
| Cain, A. R. R., M.A., B.M., B.CH. (Oxon), D.OBST. R.C.O.G., D.C.H., M.R.C.P. (London). | Paediatric Registrar, Southmead Hospital. |
| Das, A. M., M.B., B.S. (Bihar), M.S. (Bihar), F.R.C.S. (Edin.). | Registrar in Thoracic Surgery, Frenchay Hospital. |
| Faint, Sheila, S., M.B., CH.B. (Bristol). | Registrar in Pathology, Frenchay Hospital. |
| Gibbon, R. H., M.B., B.CHIR., (Cantab), D.M.R.D. (London). | Senior Registrar in Radiology, Southmead Hospital. |
| Gibbs, Irene, E., B.SC. (St. Andrews), M.B., CH.B. (Bristol). | Clinical Assistant in Paediatrics, Southmead Hospital (3 sessions). |
| Gorst, J. A., M.B., B.S. (Lond.), D.P.M. | Assistant Psychiatrist (S.H.M.O. grade), Glenside Hospital. |
| Halford, M. E., M.D., (London). M.R.C.P. (London). | Consultant Pathologist, Weston-super-Mare General Hospital. |
| Heath, I. D., M.B., CH.B. (Edin.). | Medical Registrar, Cossham-Frenchay Hospital Group. |
| Hill, B. W., M.B., B.CH., (Bristol). | Clinical Assistant in Dermatology, Cossham Hospital (1 session). |
| Mahoney, M. P., M.B., B.CH. (Wales). | Medical Registrar, Southmead Hospital. |
| Neale, R. D., M.B., CH.B. (Bristol), D.P.M. | Assistant Psychiatrist, Barrow Hospital. |
| Noble, J. G., L.R.C.P., L.R.C.S. (Edin.) L.R.F.P. & S. (Glasgow), D.P.M. | Assistant Psychiatrist, (S.H.M.O. grade), Stoke Park Group. |
| Stephenson, J. C., L.D.S.R.C.S., (Eng.), B.D.S. (Lond.), D.ORTH.R.C.S., F.D.S.R.C.S. | Consultant Orthodontist, United Bristol Hospitals/Bath Clinical Area. |
| Wethered, H. H., B.A., M.B., B.CH., (Oxford). | Clinical Assistant in Dermatology, Southmead Hospital (2 sessions). |

### *NORTH GLOUCESTERSHIRE*

| | |
|---|---|
| Cardale, N. D., M.B., CH.B. (Bristol). | G.P. in Orthopaedics and General Surgery, Lydney and District Hospital (2 sessions). |

| | |
|---|---|
| Davis, R. E., M.A. (Cantab), M.B., B.S. (London), F.F.A.R.C.S. (Eng.). | Consultant Anaesthetist, North Gloucestershire Clinical Area. |
| Hardikar, S. M., M.B., B.S. (Poona). | Registrar in Orthopaedic & Traumatic Surgery, Cheltenham General Hospital. |
| Khan, G. Ali., M.B., B.S. (Karachi). | Registrar in Orthopaedic & Traumatic Surgery, Gloucestershire Royal Hospital. |
| Khan, M. A., M.B., B.S. (Lahore). | Registrar in Geriatrics & General Medicine Gloucestershire Royal Hospital. |
| Miller, J. S., M.B., B.S. (London), D.A., F.F.A.R.C.S. | Consultant Anaesthetist, North Gloucestershire Clinical Area. |
| Dalrymple, J. O., M.B., B.S. (London), D.OBST. R.C.O.G. | Surgical Registrar, Bath Group of Hospitals. |
| Desai, S. K. H., M.B., B.S. (Baroda). | Anaesthetic Registrar, Bath Group of Hospitals. |
| Loxdale, Hilary, B.M., B.CH. (Oxon), D.OBST. R.C.O.G., D.O. | Clinical Assistant in Ophthalmology, Bath Eye Infirmary (2 sessions). |
| Pilton, D. W., M.B., CH.B. (Bristol).<br>Wheeler, B. R., M.B., B.S. (London). | Clinical Assistants in Orthopaedic and Traumatic Surgery, Royal United Hospital Bath (1 session each). |
| Weller, S. D. V., M.D., (London), M.R.C.P., D.C.H. | Consultant Paediatrician, Bath Clinical Area. |

### *SOUTH SOMERSET*

| | |
|---|---|
| Gupta, P. K., M.B., B.S. (Calcutta). | Anaesthetic Registrar, Taunton and Somerset Hospital. |
| Lister, H.K.N., M.A., M.R.C.S., L.R.C.P. | Clinical Assistant in Anaesthetics, Minehead and West Somerset Hospital (1 session). |
| Royston, N. J. W., M.R.C.S., D.C.H., M.R.C.P. | Consultant Paediatrician, South Somerset Clinical Area. |

### *DEVON AND EXETER*

| | |
|---|---|
| Emami-Ahari, S. Z., M.D. (Teheran). | Registrar in Orthopaedic and Traumatic Surgery, Torbay Hospital/Princess Elizabeth Orthopaedic Hospital. |
| Grainger, C. N., M.B., B.S. (London), M.D. (London), D.C.H. (Eng.). | Clinical Assistant in Dermatology, Royal Devon and Exeter Hospital (1 session), re-appointed. |
| Hall, G. H., B.SC., M.D., M.R.C.P. (London). | Consultant Physician, Devon and Exeter Clinical Area. |
| Ling, R. S. M., M.A., B.M. (Oxon), F.R.C.S. (Eng.). | Consultant Orthopaedic Surgeon, Devon and Exeter Clinical Area. |
| Rub, A., L.M.F. (East Pakistan), M.B., B.S. (Dacca). | Registrar in Psychiatry, Exe Vale Hospital. |
| Munks, A. W., M.B., B.S. (London). | Clinical Assistant in Orthopaedic Surgery, Stratton Hospital (1 session). |

### *PLYMOUTH*

| | |
|---|---|
| Barnett, A. M., M.A. (Camb.), M.R.C.S., L.R.C.P. | Ophthalmologist, Plymouth Clinical Area (S.H.M.O. grade). |
| Brindle, J. M., M.A., B.M., B.CH., D.M.R.T., F.F.R. | Consultant Radiotherapist, Plymouth Clinical Area. |
| Gall, W. J., M.B., CH.B. (Bristol), F.R.C.S. (Eng.). | Consultant Surgeon, Plymouth Clinical Area. |
| Inman, M. T., M.B., B.S. (London), D.OBST.R.C.O.G., D.C.H. | Anaesthetic Registrar, Plymouth General Hospital. |
| Ross, D. N., M.D., (Glasgow), M.R.C.P. (Glasgow). | Consultant Geriatrician, Plymouth Clinical Area. |
| Wood, J., M.B., CH.B., (Birmingham). | Psychiatric Registrar, Moorhaven Hospital. |

### *WEST CORNWALL*

| | |
|---|---|
| Jaco, N. T., B.SC., M.A., B.M., B.CH., D.C.H., M.R.C.P. (London). | Consultant Paediatrician, West Cornwall Clinical Area. |

*Printed by*

J. W. Arrowsmith Ltd., Bristol

